# THE EVOLUTION OF AGHE AS A GLOBAL LEADER IN EDUCATION ON AGING: HOW, WHY, AND WHAT’S NEXT

**DOI:** 10.1093/geroni/igz038.1577

**Published:** 2019-11-08

**Authors:** Margaret A Perkinson

**Affiliations:** Univ. of Hawaiʻi at Manoa, Honolulu, Hawaii, United States

## Abstract

The scope of AGHEʻs responsibility to gerontology and geriatrics extends worldwide, as reflected in its tag line, “Global Leaders in Education on Aging.” Optimal responses to worldwide demographic transitions can only come from persons well-versed in the dimensions of aging and trained and globally situated to translate that knowledge into effective and culturally-appropriate solutions. This presentation reviews the evolution of AGHE’s role in initiating and fostering global networks of educators in gerontology and geriatrics, including collaborative efforts with major international organizations (e.g., WHO, UN, IAGG) to increase the visibility and appreciation of aging-related issues among world leaders; sponsoring national and international meetings to promote exchange of ideas and refinement of teaching methodologies; initiating and adapting new models of gerontological training enhanced by advances in information and communication technology; and supporting world-wide cohorts of emerging scholars to assume leadership roles within the organization. Recommendations for next steps are considered.

